# Global trends in dietary micronutrient supplies and estimated prevalence of inadequate intakes

**DOI:** 10.1371/journal.pone.0175554

**Published:** 2017-04-11

**Authors:** Ty Beal, Eric Massiot, Joanne E. Arsenault, Matthew R. Smith, Robert J. Hijmans

**Affiliations:** 1 Department of Environmental Science and Policy, University of California Davis, Davis, California, United States of America; 2 Program in International and Community Nutrition, University of California Davis, Davis, California, United States of America; 3 Department of Nutrition, University of California Davis, Davis, California, United States of America; 4 Department of Environmental Health, Harvard T.H. Chan School of Public Health, Boston, Massachusetts, United States of America; Robert Gordon University, UNITED KINGDOM

## Abstract

Understanding dietary patterns is vital to reducing the number of people experiencing hunger (about 795 million), micronutrient deficiencies (2 billion), and overweight or obesity (2.1 billion). We characterize global trends in dietary quality by estimating micronutrient density of the food supply, prevalence of inadequate intake of 14 micronutrients, and average prevalence of inadequate intake of these micronutrients for all countries between 1961 and 2011. Over this 50-year period, the estimated prevalence of inadequate intakes of micronutrients has declined in all regions due to increased total production of food and/or micronutrient density. This decline has been particularly strong in East and Southeast Asia and weaker in South Asia and sub-Saharan Africa. Sub-Saharan Africa is the only region where dietary micronutrient density has declined over this 50-year period. At the global level, micronutrients with the lowest levels of adequate estimated intake are calcium, iron, vitamin A, and zinc, but there are strong differences between countries and regions. Fortification has reduced the estimated prevalence of inadequate micronutrient intakes in all low-income regions, except South Asia. The food supply in many countries is still far below energy requirements, which suggests a need to increase the availability and accessibility of nutritious foods. Countries where the food energy supply is adequate show a very large variation in dietary quality, and in many of these countries people would benefit from more diverse diets with a greater proportion of micronutrient-dense foods. Dietary quality can be improved through fortification, biofortification, and agricultural diversification, as well as efforts to improve access to and use of micronutrient-dense foods and nutritional knowledge. Reducing poverty and increasing education, especially of women, are integral to sustainably addressing malnutrition.

## Introduction

Assuring all people have access to sufficient and healthy food remains one of the world’s pressing challenges. Undernourishment (hunger) has been declining but remains very high at about 11% of the global population [1]. Moreover, poor quality diets can lead to micronutrient deficiencies, especially in populations with low total food intake. Such “hidden hunger” can cause pregnancy complications and child growth failure, increase susceptibility to disease, and impair cognitive development [2]. Meanwhile, an increasing part of the world population consumes excessive amounts of food, which is associated with increased incidence of obesity and risk of diabetes and heart disease [3]. The ideal diet is diverse and adequately dense in micronutrients to meet requirements without excess energy intake. To improve access to healthy foods and reduce undernutrition and overnutrition, it is essential to understand how the food supply relates to these changing forms of malnutrition.

Most food security indices rely solely on energy availability or food diversity scores. Assessing energy availability only addresses hunger. Food diversity scores depend on food frequency questionnaires that do not measure the quantity of each food category consumed, and they do not adequately account for the varying quality of different food groups. Recently, studies have used national Food Balance Sheets (FBSs) from the United Nations (UN) Food and Agriculture Organization (FAO) to estimate national per capita micronutrient supplies. However, these studies have been limited to either select countries [4–6] or select minerals (calcium, zinc, and magnesium) and years (1992–2011) [7,8]. Smith et al. [9] conducted a comprehensive analysis estimating national per capita nutrient supplies over the past 50 years, but they did not adjust for bioavailability of nutrients or estimate intakes and requirements.

We expanded on methods by Arsenault et al. [6] by including additional countries and micronutrients; the impact of fortification; regional variation in FAO production, import, and export (PIE) data; estimating calcium in drinking water; estimating bioavailability of iron, calcium, and zinc in the food supply; and by creating indices to characterize dietary quality and micronutrient supplies.

## Methods

To characterize global trends in dietary quality and micronutrient supplies, we combined national FBSs from the FAO (http://www.fao.org/faostat/), UN population data, multiple food composition tables, and nutrient intakes and requirements data to estimate the prevalence of inadequate intakes of 14 micronutrients for all countries and years between 1961 to 2011. For each country-year we estimated the prevalence of inadequate intake from the distribution of the availability of each micronutrient and the population-weighted Estimated Average Requirements (EARs). For each country-year, we also created an estimated Prevalence of Inadequate Micronutrient Intake Index (PIMII; average estimated prevalence of inadequate intake for 14 micronutrients) and a Micronutrient Density Index (MDI; average micronutrient density of the food supply based on 14 micronutrients, each capped at the 2011 global population-weighted Recommended Dietary Allowance [RDA]).

### Food supply data

The FAO collects annual data on production, import, and export of agricultural products to produce national level FBSs. Secondary data and expert opinion are used to account for refuse and retention factors and provide an estimate of per capita daily energy availability (PCDEA) for 94 food categories. FBSs do not account for local or small-scale agricultural production and harvest of wild plants. This likely increases uncertainty, mostly in developing countries and particularly in rural areas where many people are dependent on subsistence agriculture and wild foods. Variation in household food waste is likely to reduce certainty in developed countries, where more food is wasted at the household level. An important consideration is that national food supplies are unevenly distributed, and they do not capture the coexistence of overconsumption and underconsumption within countries. FBS data was publicly available for each year from 1961 to 2011 at the time of writing.

Some of the 94 food categories contain many products with varying micronutrient content. We disaggregated these categories, excluding seafood (see below) using FAO production, import, and export (PIE) data for 269 food categories, and refuse and retention factors from the United States Department of Agriculture (USDA) [10,11], adjusting as necessary for missing values and for estimated differences in refuse between the United States and low-income countries. Disaggregated data within seafood categories were inconsistent and could not be used to increase precision.

PIE data were erratic between years [12]. For example, *Sweetened Condensed Milk* was inconsistently listed under different food categories, such as *Condensed Milk* or *Raw Milk*. For this reason, we averaged the PIE data from 1961 to 2011 by continent. This partially corrected the initial limitation of having broadly aggregated FBS categories, by allowing regional variation within the 94 food categories. For example, *Vegetables*, *Other* contained almost all types of vegetables, many of which varied substantially between continents and by micronutrient content, such as pumpkin and cabbage, which are high and low in β-carotene, respectively.

To account for calcium in the water supply, we assumed a fixed average individual daily water intake of 1.7 L from solely water sources, with a calcium concentration of 42 mg/L [13]. These values were calculated based on the following information. Average daily water requirements in the United States are 2.9 L for adult males and 2.2 L for adult females [14]. On average, approximately 0.8 liters of water are consumed daily per person through foods [14]. We used the average of the median calcium concentrations for surface water (36 mg/L) and groundwater (48 mg/L) to reach the calcium concentration of 42 mg/L [14]. Calcium from water contributed on average 11% (5% SD) of national calcium supplies. While iron supplies in groundwater can be substantial in some places, such as Bangladesh [15], there is insufficient data in most countries and regions to make general estimates of iron from water supplies.

We used data compiled by Smith et al. [9] to produce estimates of the amount of fortified micronutrients in national food supplies based on national fortification policies documented by the Food Fortification Initiative and within national mandatory and voluntary guidelines. When a range of fortification amounts was provided, the lower bound was chosen, since some loss of micronutrients is expected to occur through processing, transport, and storage [9]. Since there was no reliable information on whether and, if so, when fortification became enforced in each country, we chose to only include estimates of fortified micronutrients for the most recent year (2011). We included estimates of the prevalence of inadequate micronutrient intakes and the PIMII for 2011 with and without including the contribution of fortification. No data on micronutrient supplementation was included.

### Food composition tables

The USDA National Nutrient Database for Standard Reference, provided the majority of the food composition data [11]. Additional food composition databases were used to fill in particular foods or micronutrients: The West African Food Composition Table [16] was used for cassava, fonio, sorghum, sweetpotato (pale yellow, yellow, and deep yellow), and camel milk. The WorldFood International Minilist [17] was used for red palm oil (RPO) and camel meat. Phytate values were obtained from the Composite Nutrient Database [18], which uses mainly the International Minilist, but also the Nutrient Data System for Research [19]. Wessells et al. [18] used equal weighting within FBS categories, so we adjusted phytate values to correspond to our weighting, described below.

We chose 469 specific foods from the food composition tables (FCTs) to match the 269 PIE and seafood categories (S2 Dataset). Since the proportion that each individual food was allotted within PIE categories was static, we attempted to choose weighting that represented the average combination of foods consumed at the global level, using available data and informed estimates. When possible, we chose unfortified foods and made estimates of fortification separately. We selected foods in the form they are most commonly consumed (e.g., cooked meat and raw fruit), including both raw and cooked versions for foods that are commonly consumed both cooked and raw (e.g., certain leafy greens and carrots). We also included different preparation methods (e.g., boiling and grilling).

The variety of sweetpotato (orange-fleshed or white-fleshed) and type of palm oil (RPO or refined) strongly affects the β-carotene content, the precursor to vitamin A. Based on dietary intake surveys and food frequency questionnaires from the Demographic and Health Surveys, we estimated that half the palm oil consumed in Africa was RPO, which contains high amounts of β-carotene. We assumed the palm oil consumed by all other regions was refined palm oil, which contains practically no β-carotene. After reviewing FCTs from various countries and regions, we estimated that half the sweetpotato consumed in the Americas is orange-fleshed. Sweetpotato consumed in all other regions was assumed to be white-fleshed.

### Micronutrient requirements

Estimated average requirements (EARs) were obtained from the Institute of Medicine (IOM) [20–24] for all nutrients except zinc, iron, and calcium. EARs for zinc were calculated using the Miller Equation [25], which adjusts for zinc bioavailability by creating physiological EARs that account for the amount of phytate in the diet, which prevents absorption by binding to zinc. To better represent international populations and allow different categories of bioavailability, EARs for iron (except pregnant women) were derived from the joint FAO/World Health Organization (WHO) Recommended Nutrient Intakes (RNIs) [26] by dividing by published conversion factors [27]. Iron EARs for pregnant women were obtained from the IOM [22] and adjusted for bioavailability since they are based on 18% absorption, unlike the WHO/FAO recommendations which provide RNIs for various absorption levels.

We classified each country-year to one of five levels of bioavailability (5%, 7.5%, 10%, 12.5%, or 15%) based on the total amount of iron, proportion of iron that is heme iron, and our estimate of non-heme bioavailability of the food supply. To estimate the proportion of iron that is heme iron for each country-year, we used reported values for different animal meats: bovine (65%), pork (39%), chicken and fish (26%), lamb and mutton (72%), and all other meats (40%) [28,29]. We assumed a fixed heme iron absorption of 25% [30], but absorption of heme iron is known to vary from 10% in iron replete to 40% in iron deficient individuals [31].

We used the three strongest dietary predictors identified by Reddy et al. [32] to estimate absorption of non-heme iron: the amount of phytate, animal tissue, and vitamin C consumed during a meal. Phytate binds to iron, preventing absorption, animal tissue increases absorption, and to a lesser extent, vitamin C increases absorption, particularly in the absence of animal tissue [32]. While FBSs do not contain single-meal estimates, we decided including these predictors of non-heme iron bioavailability would improve estimates of average per capita daily iron supplies, even with uncertainty in whether they were consumed with non-heme iron meals. We calculated the total daily per capita amounts of phytate, animal tissue, and vitamin C and grouped values into 11-quantiles for each by country-year, and standardized these to values between 0 and 1. To estimate the percentage of non-heme iron absorption for each country-year we applied the following equation:

Abs=18(0.4A+0.4(1–P)+0.2C)

where *Abs* is the percentage of non-heme iron absorbed, *A* is the animal tissue value, *P* is the phytate value, and *C* is the vitamin C value. We assumed a two-fold greater effect of animal tissue and phytate than vitamin C on absorption. This equation allows a maximum non-heme iron absorption value of 18%, which corresponds to the meal with the highest adjusted non-heme iron absorption percentage in the study by Reddy et al. [32]. The estimates of non-heme iron absorption (between 0–18%) were combined with the static heme iron absorption value of 25% based on the proportion of heme and non-heme iron supplies to assign each country-year to one of the five groups of bioavailability: 5%, 7.5%, 10%, 12.5%, or 15%.

For calcium, we derived temporary EARs (for the purpose of adjusting calcium supplies according to animal protein availability) from *standard* RNIs and *hypothetical* RNIs suggested by the FAO/WHO expert consultation [26]—which better represent international populations and allow for the creation of different categories of bioavailability—by dividing by conversion factors [27]. Since consumption of animal protein increases urinary calcium excretion and appears to increase risk of hip fracture in individuals with low calcium intake [33], we created three different categories of bioavailability: populations with a per capita availability of (*A*) less than 40 grams of animal protein, (*B*) between 40 and 60 grams, and (*C*) 60 grams or greater. Country-years in group *A* were given temporary EARs derived from *hypothetical* RNIs [26]. Country-years in group *C* were given temporary EARs derived from *standard* RNIs [26]. Country-years in group *B* were given the average temporary EAR’s of groups *A* and *C*. Calcium supplies were then adjusted to account for differences in bioavailability depending on animal protein supplies. Temporary EARs described above were only created to adjust calcium supplies, and not final requirements, so final EARs were only affected by the national population distribution.

### Estimating micronutrient intake distributions

For each micronutrient, we estimated a population distribution around the mean estimated intake per capita derived from the FBS data by calculating a coefficient of variation (CV) of intake based on within-subject variation from published dietary intake studies [34–37] (S3 Dataset). Following Arsenault et al. [6], we assumed that micronutrient intake is normally distributed if the CV is 0.3 or lower (calcium, folate, magnesium, niacin, phosphorus, riboflavin, thiamin, vitamin B6, and zinc) and log-normally if the CV is greater than 0.3 (copper, iron, vitamin A, vitamin C, and vitamin B12). We applied this CV to all country-years to obtain a distribution of estimated micronutrient intakes for each country-year.

### Estimating the prevalence of inadequate micronutrient intake

We used the EAR cut-point method [38] to estimate the prevalence of inadequate micronutrient intakes based on these approximated micronutrient intakes. This method relies on three assumptions: the distribution of intakes varies more than the distribution of requirements; the distribution of requirements is symmetrical; and intakes and requirements are not correlated [26]. There have not been large enough experimental studies to confirm that all micronutrient intakes are normally distributed for all population groups. Moreover, there are known groups where the distribution is skewed, such as iron intake requirements for menstruating women [22]. However, we were unable to use the full probability approach to estimate the prevalence of inadequate iron intakes [22] since FBSs give no information on the distribution of usual intakes.

The population data were merged with EARs by age and sex [39]. We assumed the number of pregnant and lactating women were respectively 75% and 100% of the 0 to 1-year-old population. The population of pregnant and lactating women was divided into the three EAR age categories as follows: 25% to the 14–19 and 31–51 age groups and 50% to the 19-31-year-olds. Children under 6 months were assumed to be exclusively breastfed. Children 6–12 months were given an EAR half that of the EAR for 1-3-year-olds, to account for the introduction of complementary foods. To create a national EAR for each country-year, we summed the EARs for each age and sex subgroup, weighted by their respective population, and divided by the total population. The proportion of the population below the EAR was considered to have inadequate estimated intake.

### Indices

We calculated the estimated Prevalence of Inadequate Micronutrient Intake Index (PIMII) for each country-year by averaging, with equal weighting, the estimated prevalence of inadequate intakes of 14 micronutrients: calcium, copper, iron, folate, magnesium, niacin, phosphorus, riboflavin, thiamin, vitamin A, vitamin B12, vitamin B6, vitamin C, and zinc. We used the following equation to calculate the Micronutrient Density Index (MDI) for each country-year:

$$MDI=\frac{1}{n}\sum_{i=1}^{n}min\{1,(\frac{M_{i}}{E}/\frac{{RDA}_{i}}{EER})\}$$

where *i* is one of *n* micronutrients (14 in our case), *EER* is the 2016 global population-weighted per capita daily Estimated Energy Requirement for a moderately active individual (2,498 kcal) [21], *M*_*i*_ is the per capita daily micronutrient availability, *RDA*_*i*_ is the 2011 global population-weighted RDA [20,22–24], and *E* is the per capita daily energy availability. Any micronutrient with a density higher than 1 (the *RDA*_*i*_) was capped at the *RDA*_*i*_ to prevent a very high availability of any micronutrient from overly influencing the MDI. Otherwise, a very high availability of one micronutrient could compensate for a shortage of other micronutrients.

## Results

**Fig 1:**
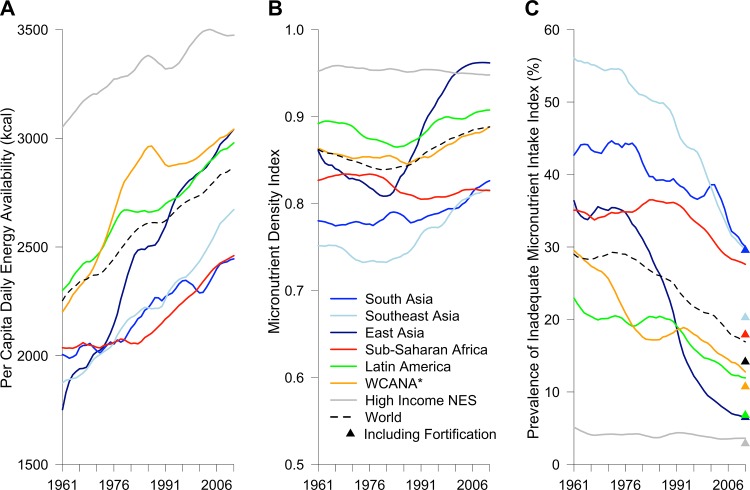
Global trends in national food supplies from 1961 to 2011. (A) Per capita daily energy availability, (B) Micronutrient Density Index, and (C) Estimated Prevalence of Inadequate Micronutrient Intake Index for the world and 7 regions. Trends are based on a five-year moving average. Color-coded triangles represent the value for each region in 2011 when including the contribution of fortification. *WCANA stands for West Central Asia and North Africa; “High Income NES” stands for High Income countries not elsewhere specified (Australia, Canada, New Zealand, United States, and Western Europe).

Globally, national per capita daily energy availability (PCDEA) has increased steadily since 1961, except in South Asia (which is largely impacted by the population of India), where it did not begin to increase substantially until 1981, and sub-Saharan Africa, where it did not begin to increase substantially until 1985 (Fig 1A). The World mean PCDEA was about 2,250 in 1961 and 2,850 in 2011 (Fig 1A). PCDEA was lowest in East Asia in 1961, but has increased considerably due to economic growth in China, surpassing the world average in 1994 (Fig 1A). In High Income countries not elsewhere specified (High Income NES)—which includes Australia, Canada, New Zealand, the United States, and Western Europe—PCDEA plateaued at 3,500 kcal in 2005 and has slightly decreased since (Fig 1A).

**Fig 2:**
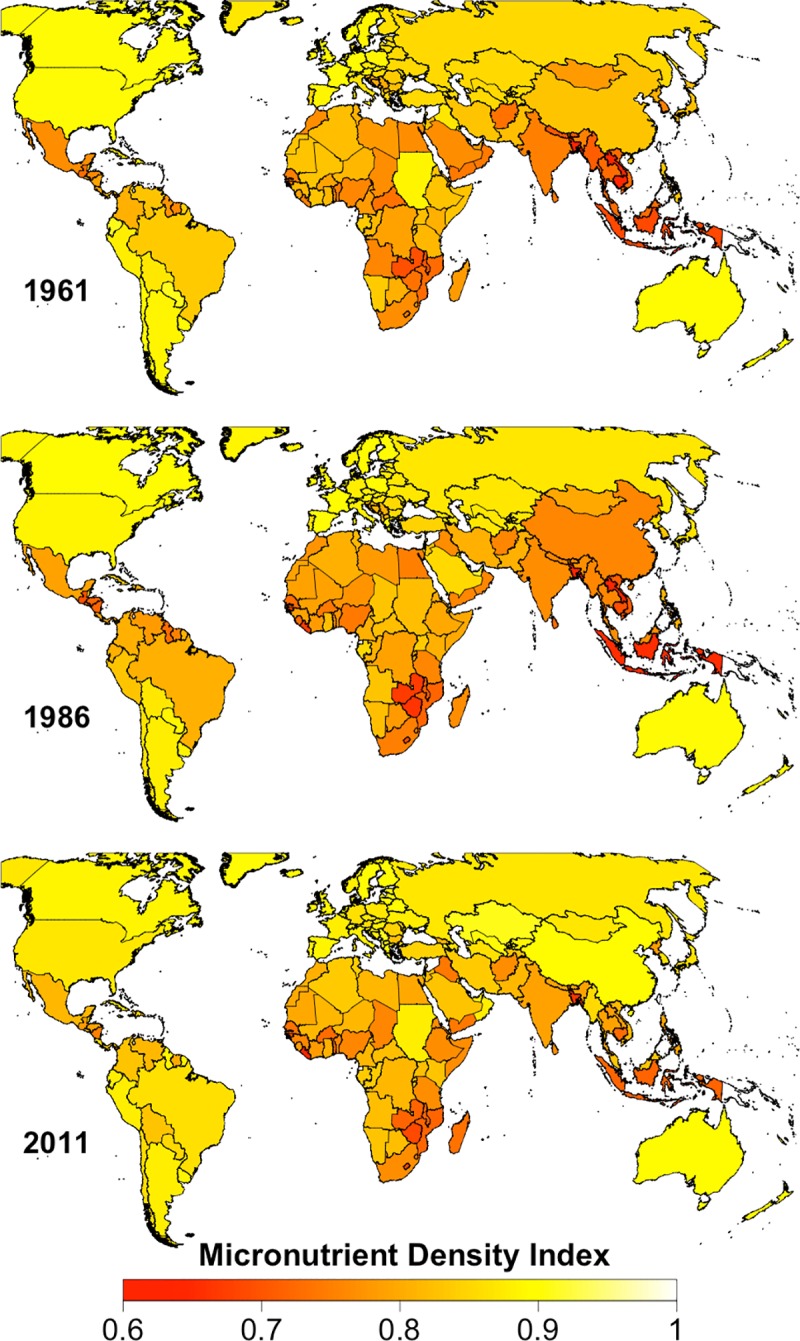
Country-level Micronutrient Density Index for the years 1961, 1986, and 2011. Values for countries with missing data (Libya and DRC) were estimated based on the values of neighboring countries.

From 1961 to the 1980s the MDI declined in most regions, particularly in Latin America, and in East and Southeast Asia (Figs 1B and 2). The MDI has risen in most regions since the 1980s, but this increase in the MDI appears to be tapering off in East Asia and Latin America (Figs 1B and 2). The MDI declined dramatically from 1979 to 1993 in sub-Saharan Africa, due to the increased availability of lower-micronutrient density grains (rice, maize, and wheat) and vegetable oils and decreased proportional availability of pulses, dairy products, meat, nuts and seeds, and fruit (Fig 1B and S1–S3 Figs). Throughout most of this period, Southeast Asia had the lowest MDI for any region, largely because of the high proportion of white rice in the diet and low amount of animal-source foods, but since about 2000, it has had a similar MDI to that of sub-Saharan Africa and East Asia (Fig 1B and S4 Fig). In 2011, these three regions had a far lower MDI than the rest of the world (Figs 1B and 2). After 1983, the MDI of East Asia improved dramatically, and since 2003 it has had the highest value of all our regions (Figs 1B and 2). The five countries with the highest MDI in 2011 (.99) were Antigua and Barbuda, Kazakhstan, Armenia, Albania, and Serbia (S1 Dataset).

**Fig 3:**
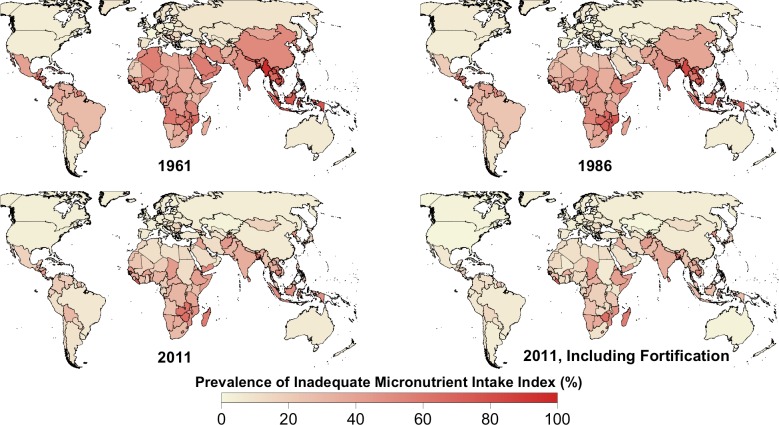
Country-level estimated Prevalence of Inadequate Micronutrient Intake Index (PIMII) for the years 1961, 1986, and 2011. For 2011, the PIMII when including the contribution of fortification is also shown. Values for countries with missing data (Libya and DRC) were estimated based on the values of neighboring countries.

**Fig 4:**
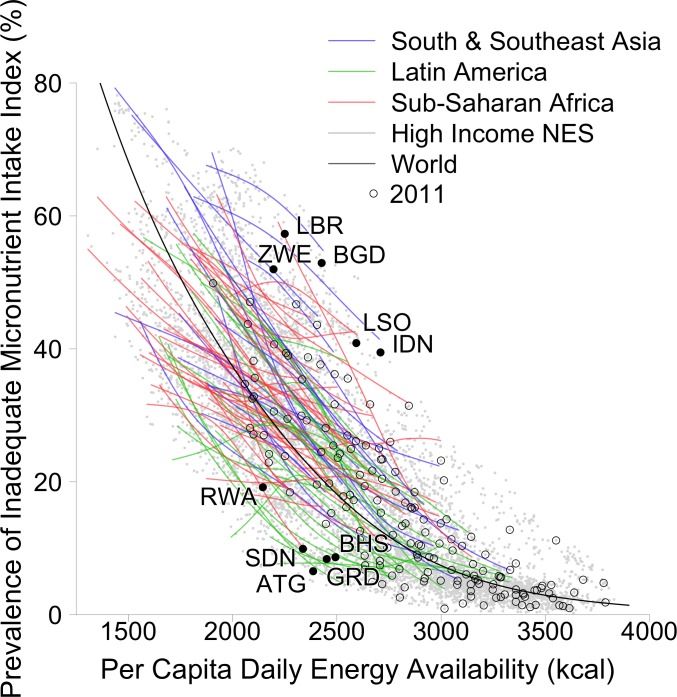
Per capita daily energy availability versus the estimated Prevalence of Inadequate Micronutrient Intake Index for all country-years, excluding fortification. Gray points represent values for every country-year between 1961 and 2011. The thick black line shows a smooth-spline model fitted with these data. Smooth spline model-derived lines for each country are also shown, color-coded by select regions. Black open circles represent values for every country in 2011. Black solid points and ISO3 codes are shown for the 5 largest country outliers in 2011 in either direction relative to the smoothed-spline model for the world: ZWE (Zimbabwe), LBR (Liberia), BGD (Bangladesh), LSO (Lesotho), IDN (Indonesia), RWA (Rwanda), SDN (Sudan), ATG (Antigua and Barbuda), GRD (Grenada), and BHS (Bahamas). “High Income NES” stands for High Income countries not elsewhere specified (Australia, Canada, New Zealand, United States, and Western Europe).

At the global level, the PIMII was above 27% from 1961 to 1983 and declined steadily to 17% (14% with fortification) in 2011 (Fig 1C). This was largely due to a rapid decline in the PIMII from 1978 to 2011 in East Asia (35% to 7%) and Southeast Asia (53% to 31% [20% with fortification]) and considerable declines in South Asia (44% to 32%) and from 1991 to 2011 in Latin America (20% to 12% [7% with fortification]), sub-Saharan Africa (36% to 28% [18% with fortification]), and WCANA (19% to 13% [11% with fortification]) (Figs 1C and 3). The ten countries with the highest PIMII in 2011 when including fortification were Liberia (57%), Bangladesh (53%), Zimbabwe (52%), Madagascar (47%), Timor-Leste (44%), Cambodia (44%), Guinea-Bissau (43%), Lesotho (41%), Mozambique (39%) and Swaziland (39%) (Fig 3 and S1 Dataset). In contrast, excluding fortification, the PIMII in 2011 was at or below 10% for Antigua and Barbuda (7%), Grenada (8%), the Bahamas (9%), and Sudan (10%), all of which had a PCDEA of less than 2,500 kcal (Fig 4 and S1 Dataset). When including fortification, the PIMII in 2011 for Kenya (8%) and Rwanda (11%) are in stark contrast to Liberia (57%) and Zimbabwe (52%), even though all four countries had a PCDEA between 2,145 and 2,250 kcal (S1 Dataset). All countries with a PCDEA above 3,000 kcal had a PIMII below 10% in 2011 when including fortification except Kiribati (20%), Mauritius (11%), and Morocco (11%) (S1 Dataset).

**Fig 5:**
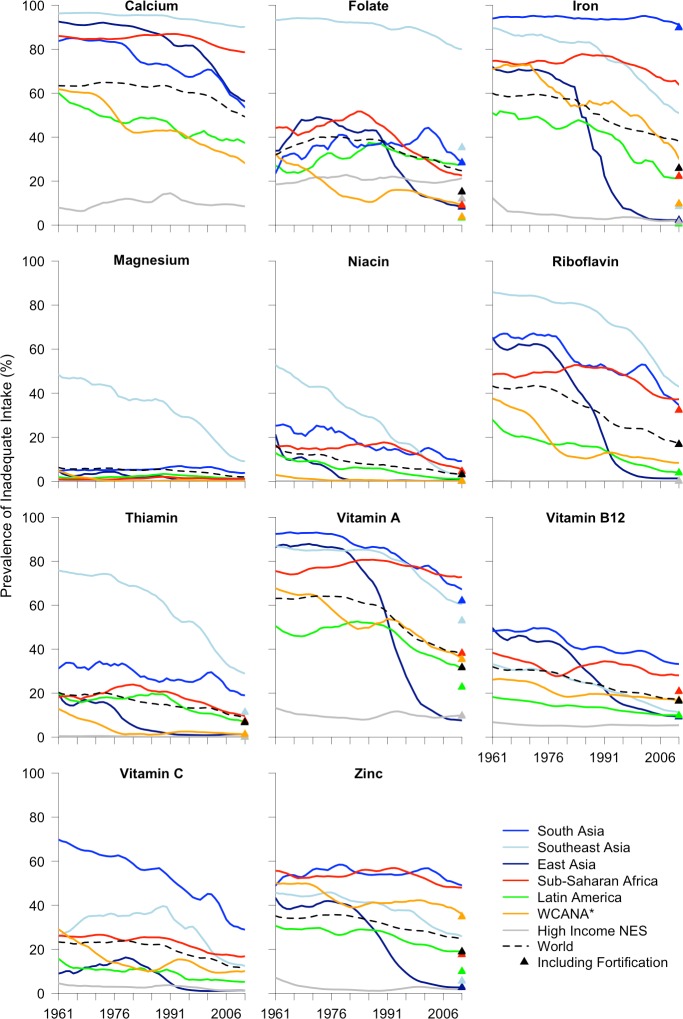
Estimated prevalence of inadequate intake of 11 micronutrients between 1961 and 2011 for the world and 7 regions. Trends are based on a five-year moving average. Color-coded triangles represent the value in 2011 when including the contribution of fortification. *WCANA stands for West Central Asia and North Africa; “High Income NES” stands for High Income countries not elsewhere specified (Australia, Canada, New Zealand, United States, and Western Europe).

**Fig 6:**
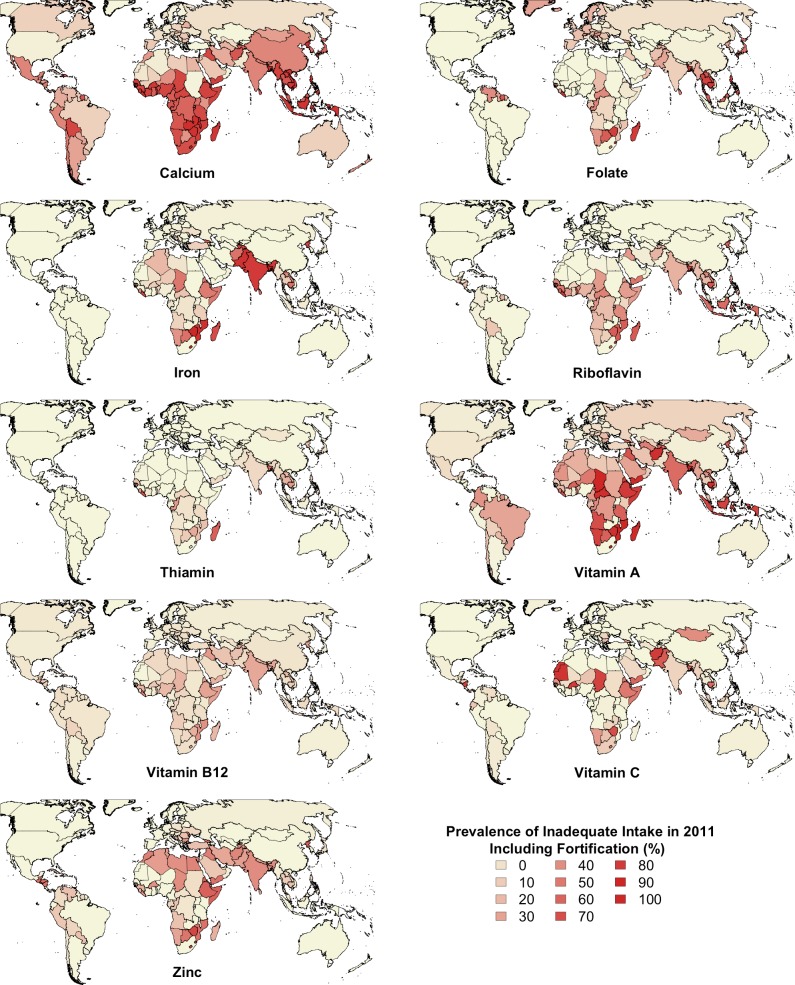
Country-level estimated prevalence of inadequate intake of 9 micronutrients in 2011, including the contribution of fortification. Values for countries with missing data (Libya and DRC) were estimated based on the values of neighboring countries.

The micronutrients with the highest estimated prevalence of inadequate intake globally in 2011 were calcium, iron, vitamin A, folate, zinc, riboflavin, and vitamin B12 (Figs 5 and 6). Southeast Asia had the highest estimated prevalence of inadequate intake of calcium, folate, magnesium, riboflavin, and thiamin for all years between 1961 and 2011 (Fig 5). When including fortification, South Asia had a higher estimated prevalence of inadequate intake of thiamin in 2011 (Fig 5). South Asia had the highest estimated prevalence of inadequate intake of iron and vitamin C for all years between 1961 and 2011 and most years for vitamin A, vitamin B12, and zinc (Fig 5). Excluding “High Income NES” countries, sub-Saharan Africa had the lowest reduction in the estimated prevalence of inadequate intake of most micronutrients and in 2011 maintained the highest estimated prevalence of inadequate intake of vitamin A and second highest for calcium, iron, vitamin B12, zinc, vitamin C, and niacin (Fig 5). However, when including fortification, South and Southeast Asia both had a higher estimated prevalence of inadequate intake of vitamin A in 2011 (Fig 5). In Latin America, the micronutrients with the highest estimated prevalence of inadequate intake in 2011 were calcium, vitamin A, folate, iron, and zinc (Fig 5). When including fortification, the estimated prevalence of inadequate intake of iron and zinc reduced dramatically (Fig 5). In WCANA the micronutrients with the highest estimated prevalence of inadequate intake in 2011 were zinc, vitamin A, iron, and calcium, although when including fortification, the estimated prevalence of inadequate intake of iron reduced substantially (Fig 5). In “High Income NES” countries, the micronutrient with the highest estimated prevalence of inadequate intake in 2011 was folate, even when including fortification (Fig 5).

Fortification reduced the PIMII in 2011 by about 3% globally, nearly 10% in both sub-Saharan Africa and Southeast Asia, 5% in Latin America, and 2% in WCANA (Fig 1C). Fortification in sub-Saharan Africa greatly increased estimated intake of iron, vitamin A, and zinc, and moderately increased intake of folate, vitamin B12, and riboflavin (Fig 5). Fortification in Southeast Asia greatly increased estimated intake of folate, iron, zinc, and thiamin, and moderately increased estimated intake of vitamin A (Fig 5). Fortification in Latin America greatly increased estimated intake of folate and iron and moderately increased estimated intake of vitamin A, zinc, and thiamin (Fig 5). Fortification in WCANA greatly increased estimated intake of iron and moderately increased estimated intake of folate (Fig 5). Fortification in South Asia only moderately increased estimated intake of vitamin A (Fig 5). Fortification in many individual countries has caused a substantial reduction in the PIMII, such as Zambia (40%), Malawi (28%), Senegal (20%), Indonesia (19%), and Togo (18%) (Fig 3 and S1 Dataset).

## Discussion

Over the past 50 years, the estimated prevalence of inadequate micronutrient intakes globally has been greatly reduced through increased food supplies and/or improved micronutrient density. The reduction in micronutrient density of the food supply in sub-Saharan Africa is an exception to this trend because the proportion of micronutrient-poor grains (rice, maize, and wheat) and vegetable oils in the diet has increased at the expense of more nutritious foods. To improve the micronutrient density of the diet and increase micronutrient intakes in sub-Saharan Africa, efforts should be made to increase access to and produce the appropriate amounts of micronutrient-dense foods such as vegetables; fish, shellfish, and mollusks; pulses; dairy products; eggs; meat; and nuts and seeds.

The food energy supply (PCDEA) in many countries is still far below intake requirements; there are about 795 million people without enough food [1]. It is therefore important to continue increasing the food available in these countries, especially micronutrient-dense foods such as vegetables, pulses, and animal-source foods, as well as staples. Growth of the food supply can be supported by efforts to reduce poverty, and investments in transportation, education, and agricultural research and development, as well as policy that improves equitable access to nutritious food. In many countries where the food energy supply is adequate or nearly so, efforts to improve the quality of the food supply, such as agricultural diversification, fortification, and biofortification are warranted, as well as improved access to and use of micronutrient-dense foods. The preferred balance between such approaches will vary between countries, but the strong variation in the PIMII (by more than 50%) in countries with a PCDEA less than 2,500 kcal suggests it is possible to reduce the estimated prevalence of inadequate micronutrient intakes without producing more, by producing foods with higher micronutrient densities. This can be achieved in various ways, but usually includes increasing dietary diversity (i.e., many food groups contributing a significant proportion of the diet) and producing at least a moderate amount of animal-source foods, especially fish, shellfish, milk, and eggs.

Animal-source foods contain highly bioavailable micronutrients, particularly iron, vitamin A, calcium, zinc, and vitamin B12, which can be difficult to consume in sufficient quantities on a mostly vegetarian diet. The role and ideal quantity of animal-source foods is not well settled. Nevertheless, the demand for animal-source foods is expected to nearly double by 2050 [40], mostly from low-income countries growing in population and wealth [41], which is likely to improve micronutrient nutrition in these countries. In contrast, the amount of animal-source foods in the food supply in many wealthy countries exceeds what many consider healthy or necessary. Moreover, increased production of animal-source foods on suitable cropland may lead to increased greenhouse gas emissions and, if conventionally-raised, a demand for additional cropland to produce feed [41]. However, livestock raised on rangelands can provide environmental benefits if managed properly [41].

National PCDEA well above a population’s energy requirements may be associated with an increased risk of obesity and related diseases. More than 25% of the global population (2.1 billion) is overweight or obese, an increase of 28% in adults and 47% in children between 1980 and 2013 [42]. This highlights the importance of a highly bioavailable, micronutrient-dense diet in wealthy countries, not just low-income countries. Overconsumption is associated with increased rates of noncommunicable diseases that may be preventable or treatable through lifestyle alteration, which could greatly reduce the global disease burden. Populations with a high prevalence of obesity and overweight may benefit from decreasing the quantity of their food supply while improving dietary quality so that micronutrient requirements can be met with less energy intake.

It is possible to improve the micronutrient density of the food supply by increasing fruit and vegetable production or small-scale livestock through agricultural diversification, for example by facilitating home gardening [43]. In this context, it would be helpful to evaluate agricultural production for its nutritional production in addition to mass or energy yield [44], which could incentivize the production of more nutritious food. Programs that attempt to improve micronutrient status through increased household agricultural production are likely to be more effective when paired with nutrition and health behavior change communication [45]. Arimond et al. [46] outline five pathways for how agricultural interventions can improve nutrition: consumption of own production; increases in income; reductions in market prices; shifts in consumer preferences; and shifts in control of resources.

Fortification has reduced micronutrient deficiencies for over 80 years in developed countries [27], and we show that many regions have made substantial reductions to the estimated prevalence of inadequate micronutrient intakes through national fortification programs. Unfortunately, many low-income countries do not have fortification legislation in place, especially in South Asia. Targeted implementation of fortification programs, surveillance, and enforcement can greatly increase global micronutrient intakes. More emphasis should be placed on fortifying foods with micronutrients estimated to be in shortest supply: calcium, iron, vitamin A, and zinc. Our results show that South Asia has made little use of fortification programs and has potential to greatly increase micronutrient intakes. Implementation of fortification programs present challenges such as ensuring fortificants are consumed in the appropriate quantities (adequate while limiting risk of excessive intake), an equitable geographic reach (rural communities are often not reached), and managing nutrient losses. Additionally, fortificants do not usually include beneficial non-nutritive components such as fiber and phytochemicals.

The selection and development of staple crop varieties with high levels of micronutrients (biofortification), such as high-β-carotene sweetpotato [47] and rice [48], high-iron beans [49], and high-iron, zinc, and β-carotene cassava [50] also holds promise, particularly in countries with little scope for dietary diversification or fortification [6]. For example, large-scale effectiveness trials have shown that introducing orange-fleshed sweetpotato in settings where white-fleshed sweetpotato is commonly consumed can increase intakes of β-carotene and vitamin A status [47,51]. Plant breeders are also producing crop varieties with increased absorption-promoting substances such as vitamin C and reduced anti-nutrients such as phytate and oxalate [52]. Further, methods have been used to reduce toxins such as cyanogenic glycosides in cassava, increase shelf life of edible produce, and improve viral resistance [50]. Agronomic biofortification strategies such as mineral fertilization and improvement of soil mineral solubilization and mobilization can also be used to increase the nutrients in edible portions of crops, yet these strategies are costly and have environmental costs [52]. It has yet to be seen how large of an impact biofortified varieties can have and whether they will be widely adopted, but at the very least they can be used to help meet micronutrient needs in areas that are not adequately covered by fortification programs, such as rural communities.

It is important to note that micronutrient density measurements do not adequately address bioavailability. Food storage, processing, and cooking methods and strategic food combining can have a large impact on micronutrient retention and bioavailability of plant foods. For example, post-harvest processing of fruits and vegetables, such as maintaining cooler temperatures and short storage duration, can significantly reduce vitamin C losses [53]. Fermenting, soaking, and germinating grains and legumes has been shown to reduce anti-nutrients such as phytate and improve bioavailability of iron, calcium, zinc, and magnesium [54]. Cooking vegetables such as carrots generally increases the bioavailability of β-carotene [55]. Additionally, cooking foods high in oxalates such as spinach enhances calcium absorption, while cooking foods high in goitrogens such as kale allows for unhindered iodine absorption [54]. Consuming fat with foods greatly improves fat-soluble vitamin absorption [55,56], consuming vitamin C and heme iron increases non-heme iron absorption [32], and limiting foods high in tannins such as tea and coffee during meals allows for better non-heme iron absorption [57]. Since we used the micronutrient amounts in the available food supply without any adjustment for bioavailability to calculate the MDI, we likely overestimated the micronutrient density of plant foods compared to animal-source foods.

Food Balance Sheets (FBSs) are the only data source available to describe global, national-level spatial and temporal trends in energy and micronutrient availability. FBSs provide estimates of food availability, not consumption, though for this study we assumed they are equivalent. They also do not account for household food waste, small-scale agricultural production, and consumption of wild plants and animals. Studies have shown that FBS food supply estimates overestimate individual food intake (as measured by 24-hour recall) in high income countries, though the discrepancy varies considerably by food group [58,59]. In contrast, FBSs are more likely to underestimate intake in low- and middle-income countries due to the high prevalence of unrecorded subsistence and wild harvest foods, as well as weaker statistical institutions [60]. Additionally, there are uncertainties in food composition data, including variation in nutrient amounts between repeated samples, preparation methods, and varieties, and knowledge about what proportion each individual food item should contribute within larger food group categories in the FAO data. Uncertainties are also present in estimated intake distributions and requirement data, as this varies within and between countries over time, and may not be normally or log-normally distributed. Lastly, we used novel methods to account for the bioavailability of iron and calcium in foods supplies, and while we believe they are an improvement over making no adjustment for bioavailability, these methods have not been validated through prior studies. Nevertheless, we believe our novel method for estimating aggregate micronutrient supplies provides a metric for monitoring dietary quality in populations over time, assuming many sources of error hold relatively constant within countries. Additionally, both the PIMII and MDI may find utility in assessing the effects of adverse events (famines, droughts, conflict) or interventions (agricultural programs, food assistance) on nutritional status.

Although we made every attempt to accurately estimate the prevalence of inadequate micronutrient intakes with the available data, we caution that we were unable to quantify the uncertainty of our estimates, since nationally representative dietary intake surveys that quantify nutrient intakes are not currently widely available and are prone to underreporting [61]. In addition, factors other than dietary intake can significantly affect micronutrient status, especially infection. Furthermore, since our focus was on global and regional trends, we were unable to sufficiently account for issues of war, drought, famine, or missing data in individual countries. Results for countries that have faced these issues or are missing data, such as Sudan, Libya, and DRC, should be interpreted with caution. Nevertheless, Arsenault et al. [6] showed that the method we have used can produce similar results to nationally representative dietary intake surveys.

## Conclusion

We estimated global micronutrient supplies at the national level of eight vitamins and six minerals, their bioavailability, and prevalence of inadequate intake, including an estimated Prevalence of Inadequate Micronutrient Intake Index (PIMII) and a Micronutrient Density Index (MDI). We found that the MDI has improved over the past 50 years in most regions, but it has declined in sub-Saharan Africa. The PIMII has declined in all regions during this period—although in sub-Saharan Africa it only started to decrease in 1990—due to increased total energy supplies and/or dietary micronutrient density. Increasing the availability of food energy supplies is still necessary for countries whose energy supplies fall short of their population’s requirements, and this may reduce both hunger and micronutrient deficiencies, although efforts to increase dietary micronutrient density should also be considered.

Countries with adequate energy supplies but a high estimated prevalence of inadequate micronutrient intakes may benefit from a greater focus on increasing the nutrient density of the food supply through diversification, fortification, and biofortification; improving access to and utilization of nutrient-dense foods; and nutrition education. General approaches to reduce poverty and increase education, especially of women, are integral to sustainably addressing malnutrition in low- and middle-income countries. Our indices may be used on their own, or in conjunction with the Global Hunger Index [62] or specific anthropometric indicators such as stunting and wasting or overweight and obesity to understand spatial and temporal trends in malnutrition, dietary quality, and related health effects.

## Supporting information

S1 Dataset Impact of fortification. (CSV)

S2 Dataset Compiled food composition table items. (CSV)

S3 Dataset Intake coefficients of variation. (CSV)

S4 Dataset Micronutrient densities and prevalence of inadequate intake. (CSV)

S5 Dataset MDI and PIMII. (CSV)

S1 Fig Average global micronutrient density from 1961–2011 by select food groups. Note: Bioavailability is not considered for most nutrients. Micronutrients in animal-source foods are generally more bioavailable, which is not considered here.
(TIF)

S2 Fig Per capita daily energy availability of select food groups in sub-Saharan Africa. (TIF)

S3 Fig Proportional availability of select food groups in sub-Saharan Africa. (TIF)

S4 Fig Per capita daily energy availability of rice and animal-source foods in Southeast Asia. (TIF)

S5 Fig Country-level estimated prevalence of inadequate intake of 9 micronutrients in 2011, excluding fortification. Values for countries with missing data (Libya and DRC) were estimated based on the values of neighboring countries. Source: S4 Dataset.
(TIF)
