# Supplementary figures and images for: Global trends in dietary micronutrient supplies and estimated prevalence of inadequate intakes
Source: PLoS One. 2017 Apr 11;12(4):e0175554. doi: 10.1371/journal.pone.0175554 (PMC5388500; doi:10.1371/journal.pone.0175554)

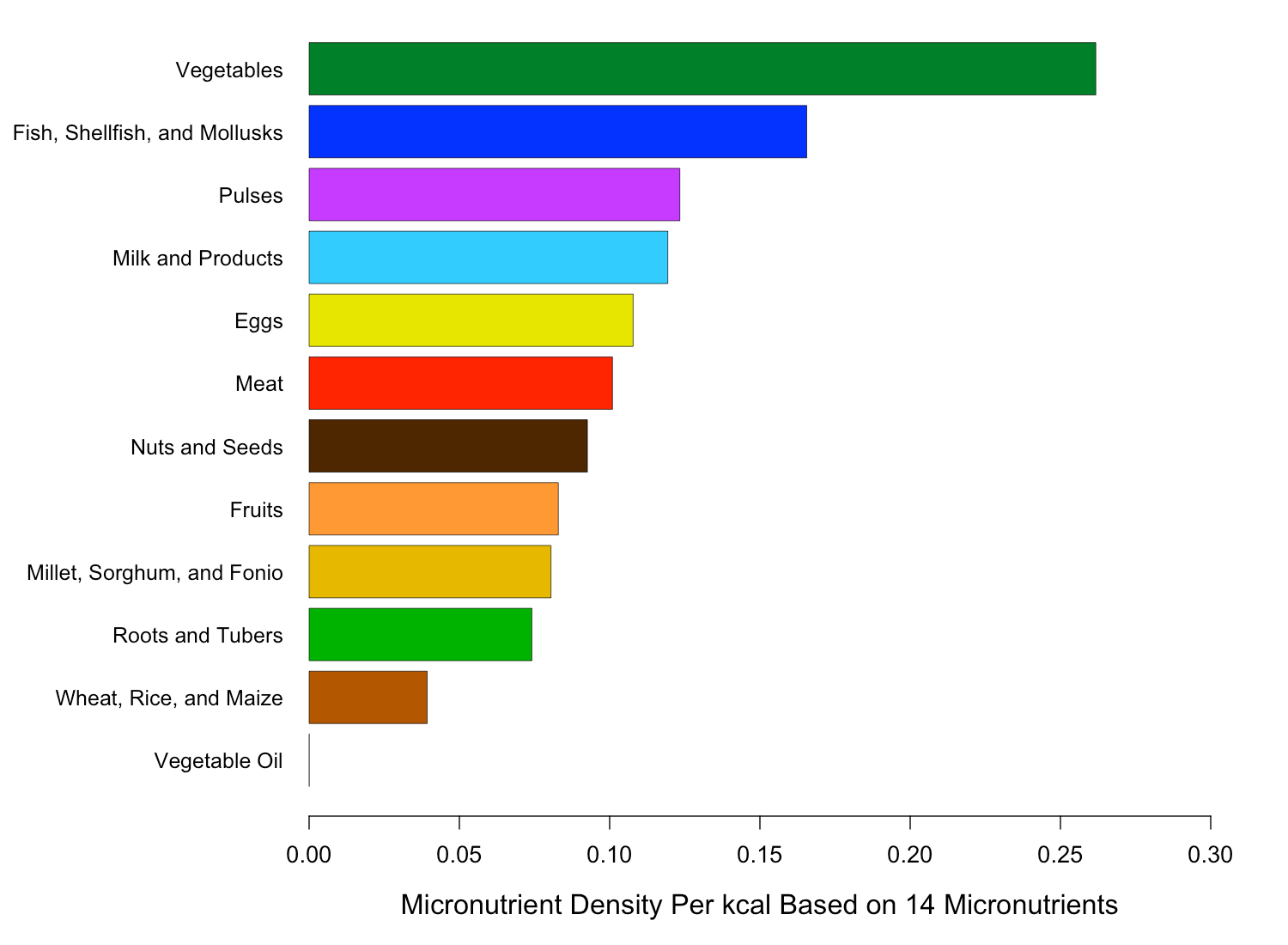

Supplement: S1 Fig — Note: Bioavailability is not considered for most nutrients. Micronutrients in animal-source foods are generally more bioavailable, which is not considered here. (TIF) [file pone.0175554.s006.tif]

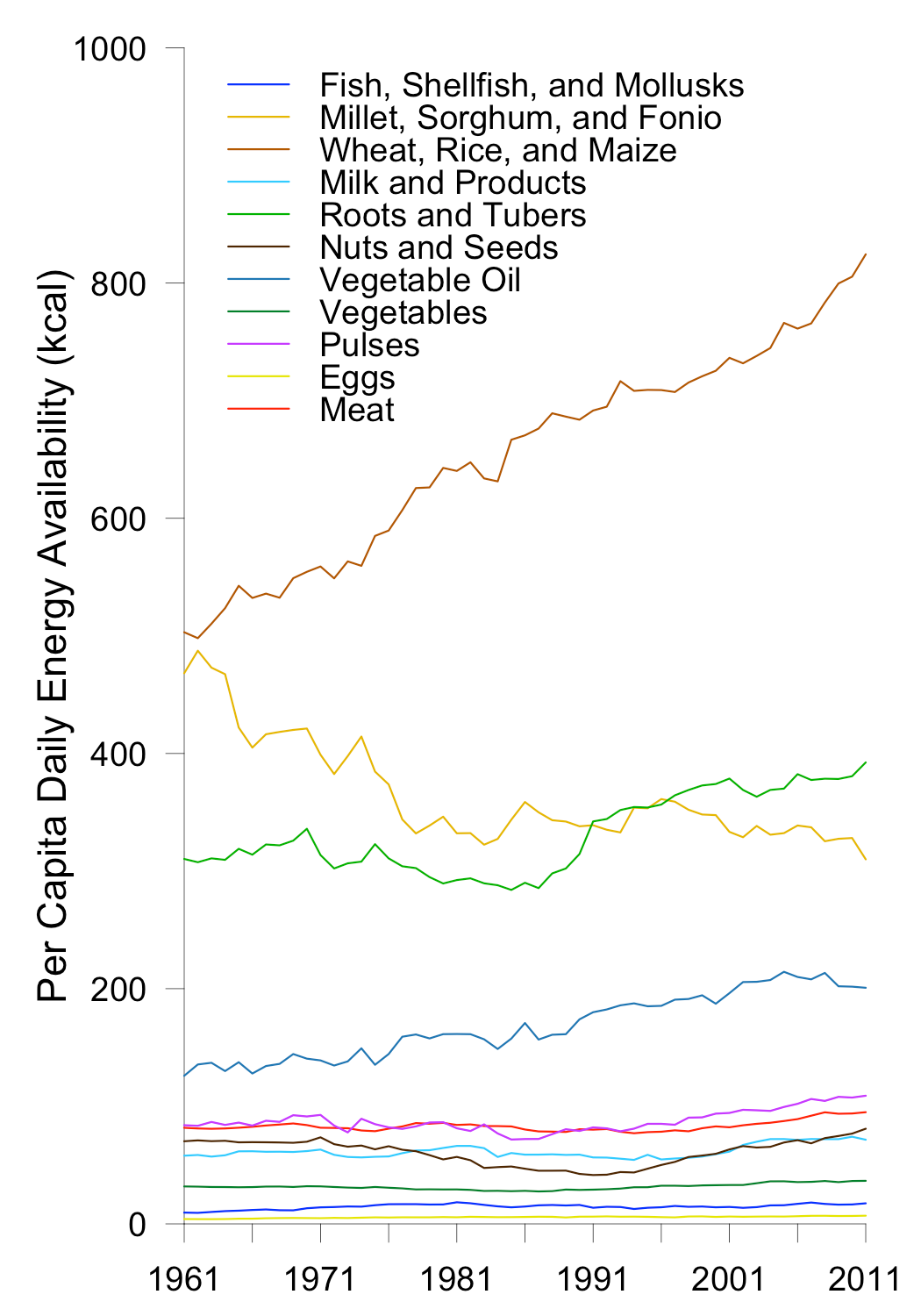

Supplement: S2 Fig — (TIF) [file pone.0175554.s007.tif]

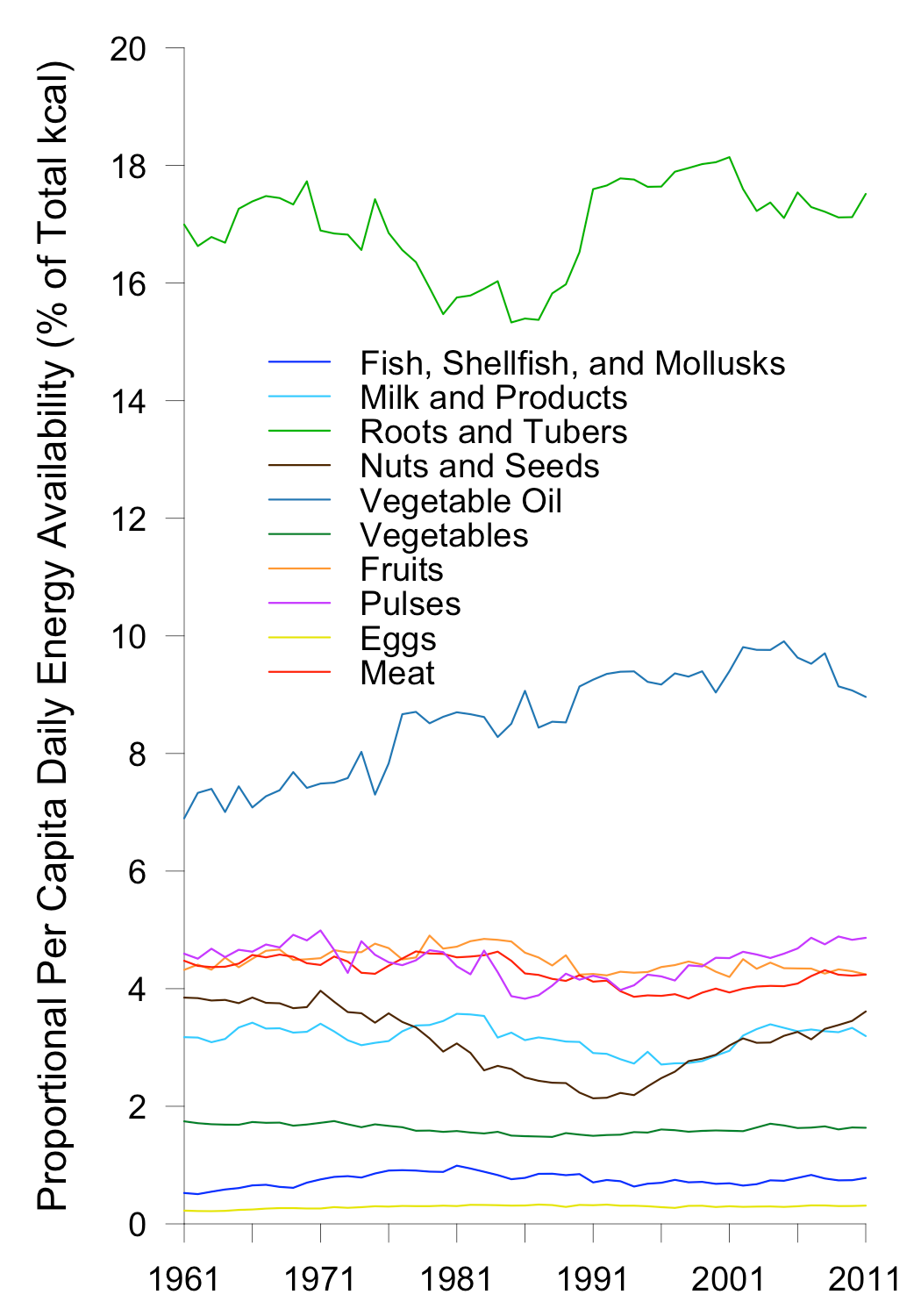

Supplement: S3 Fig — (TIF) [file pone.0175554.s008.tif]

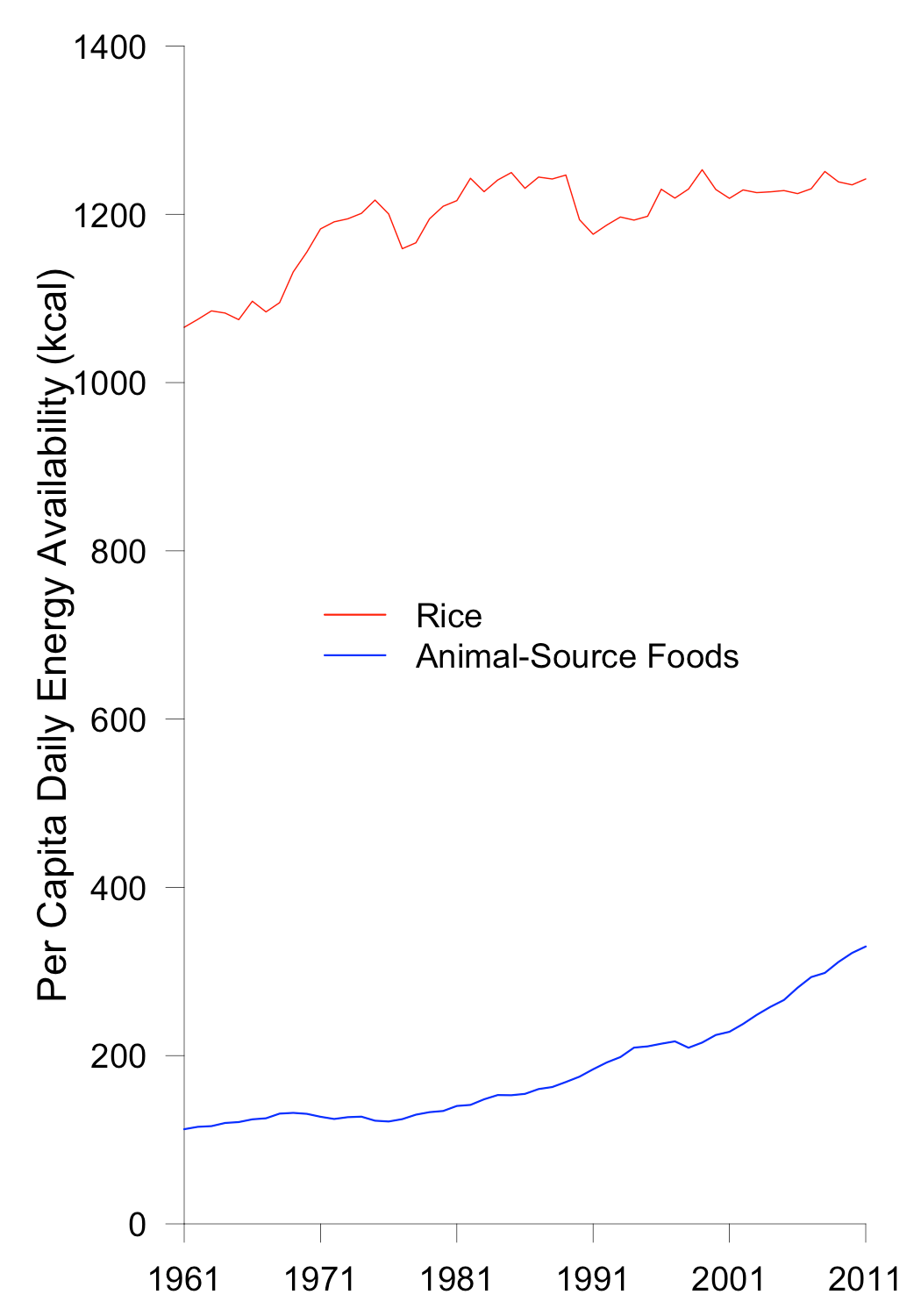

Supplement: S4 Fig — (TIF) [file pone.0175554.s009.tif]

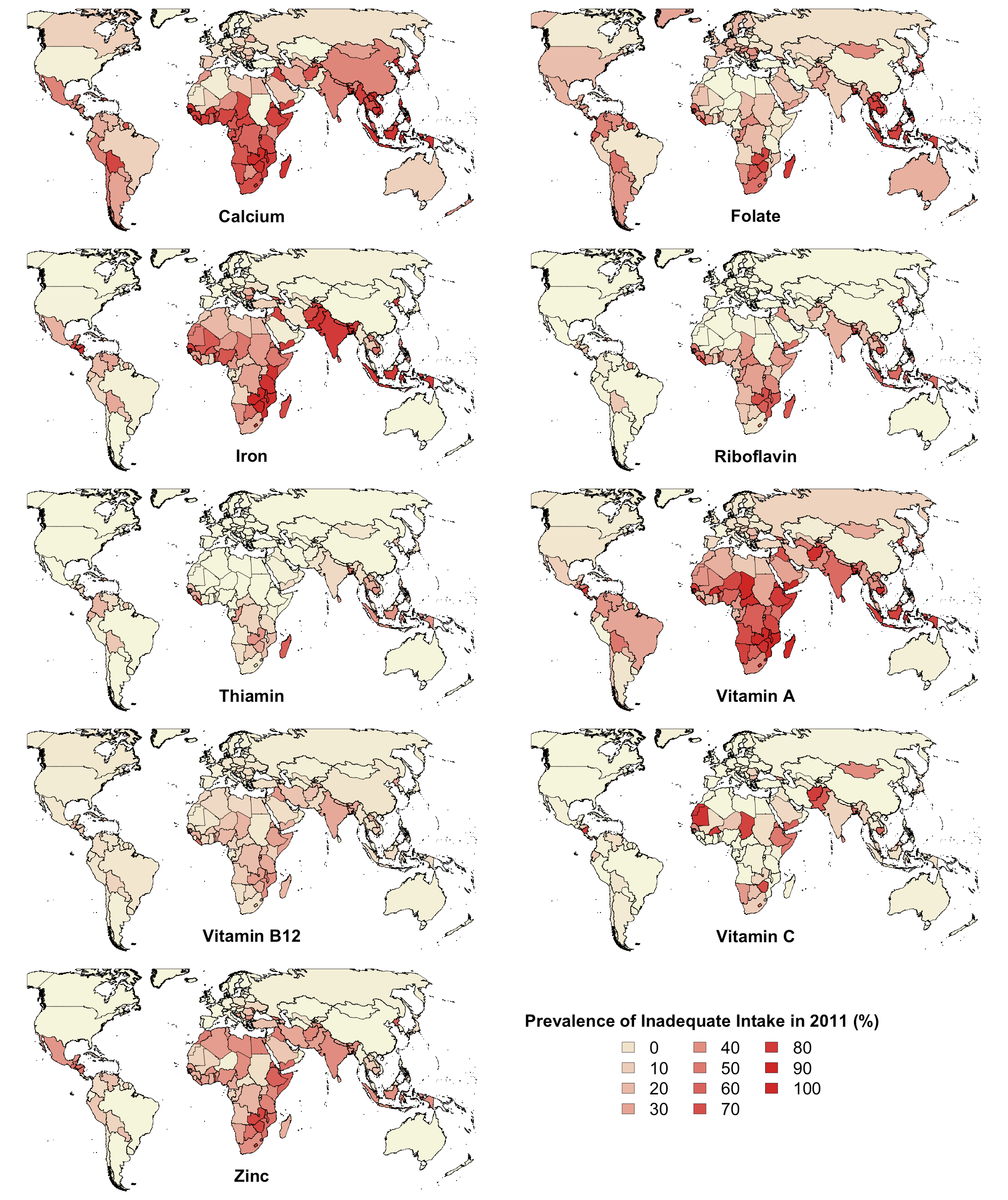

Supplement: S5 Fig — Values for countries with missing data (Libya and DRC) were estimated based on the values of neighboring countries. Source: S4 Dataset. (TIF) [file pone.0175554.s010.tif]
